# The experience of unmarried mothers raising their children in residential facilities: a phenomenological qualitative study

**DOI:** 10.1186/s12905-022-01727-9

**Published:** 2022-07-05

**Authors:** Sungjae Kim, Kyung-Sook Bang, Yeseul Jeong, Gumhee Lee, Da-Ae Shin, Misook Kim

**Affiliations:** 1 College of Nursing·The Research Institute of Nursing Science, Seoul National University, Seoul, 03080 South Korea; 2 College of Nursing, Seoul National University, Seoul, 03080 South Korea

**Keywords:** Unmarried mother, Parenting, Facilities, Qualitative research, Phenomenology

## Abstract

**Background:**

Birth outside of marriage has been gradually increasing in Korea. However, social perception of unmarried mothers is still negative, and a number of them are not accepted by their family. Therefore, the Korean government has implemented a policy to provide financial aid and communal residence to unmarried mothers who cannot raise children with their family, or afford residence. Unmarried young mothers who rely on this government policy have low economic independence and social adaptation skills. Additionally, they have a high chance of encountering numerous challenges in raising children due to their living conditions in residential facilities and social prejudice. This study was conducted to gain an in-depth understanding of the lived experience of unmarried mothers raising children in residential facilities.

**Methods:**

Data were collected through in-depth interviews with nine unmarried mothers living in residential facilities with their children. An interpretative phenomenological analysis was conducted to analyze the data.

**Results:**

The findings revealed that unmarried mothers struggled with various difficulties given the limitations of living in the facility, but attempted to navigate their uncertain future with the determination to be good mothers. Three main themes and eight sub-themes emerged: (1) adaptation to the identity of “unmarried mother”, (2) willingly undertaking the heavy burden of childrearing, (3) indispensable but insufficient supports from facilities. Participants had childrearing responsibilities, and tried to be good mothers for their children while struggling to adapt to their new identities. However, their self-doubt as a “good mother” and the absence of the child's father made them feel sorry for their child. Their daily experiences raising children and simultaneously preparing for their own independence were exhausting. The supports from the facilities were helpful but unsatisfactory and led to various psychosocial difficulties such as anxiety, depression, fear, guilty, and anger in unmarried mothers.

**Conclusions:**

Besides information and resources for parenting and independence, active approaches are needed to improve the psychological stability of unmarried mothers raising their children in facilities, and sustain a long-term socioeconomic support system. Thoughtful services tailored to mothers and children are also needed, instead of standardized services.

## Background

Birth outside of marriage is defined as birth given by mothers who were not “married” and includes those divorced, widowed, living with a partner, and unmarried [1]. Birth outside of marriage is increasing across the world, and its percentage grew from 7.4% in 1970 to 41.5% in 2018 among the OECD average of total births [1]. Although the percentage of birth outside of marriage has increased worldwide, differences between countries exist due to differing cultural backgrounds. The percentage of birth outside of marriage is high in Western cultures, which are more accepting of such phenomenon and offer institutional support. On the other hand, the percentage is extremely low in countries that have a negative perception against birth outside of marriage [1, 2]. The percentage of birth outside of marriage in Korea was 1.1% in 1981 and 2.3% in 2019, which is extremely low compared to the world average, but is continuously increasing [1], raising the need for research on families with birth outside of marriage.

In Korean society, which was traditionally influenced by Confucianism, male-dominant familism and sexual double standards are still prevalent [3]. Hence, despite the increasing rate of birth outside of marriage, the perception of pregnancy, childbirth, and childrearing in unmarried mothers is still negative. The mothers who undergo pregnancy, childbirth, and childrearing without marriage are labeled as “Mihonmo (unmarried mother)” and the perception of childrearing by unmarried mothers showed the lowest acceptance in a national awareness survey on family diversity [4]. Due to stigma and prejudice against unmarried mothers, even the parents of unmarried mothers are unable to accept their daughter’s pregnancy, and these unmarried mothers, disconnected from their own family and society, encounter hardships while being deprived of essential support needed for pregnancy, childbirth, and childrearing [4].

These unmarried mothers, who rely heavily on facilities, are mostly young mothers in their teens to early 20 s, and socioeconomically vulnerable [5]. This shows that unmarried mothers who live in residential facilities are unprepared as mothers, and that family support is poor and socioeconomically dependent. In general, unmarried mothers suffer from various difficulties due to two-sided characteristics: unplanned pregnancy and voluntary parenting. Considering this, unmarried mothers who live in residential facilities (UMiF) are likely to face more difficulties due to their special circumstances and tough conditions. Furthermore, social prejudice and discrimination against women with children out of wedlock, or through premarital sex, are prevalent in various cultures, including Korea [6–9]. These biases and discrimination can add to the various difficulties they experience [7, 8, 10] and also negatively affect the health of mothers and their children [11]. Therefore, professional attention and support for unmarried mothers living in facilities and raising children are essential for the health and well-being of vulnerable mothers and children.

Therefore, the Korean government began providing financial support and residence for unmarried mothers who could not raise their children in their family and obtain a residence [12]. There are two types of facilities for unmarried mothers: basic life support, and communal living support. The size of the facilities varies from 10 to 50 residents. Depending on the size and type of facility, the staff consist the center director, who is experienced in social welfare, assistant staff, nurses, and chefs. The basic life support facilities provide care for pregnancy, childbirth, and early childrearing (less than 6 months) for up to 1.5 years, whereas community residential support facilities provide care for living, childrearing, and the independence of unmarried mothers who decided to raise children for up to 3 years. The regulations on residential facilities only state a minimum area according to the number of residents and the conditions of essential spaces such as the kitchen, living room, and office [12]. The structure of facilities can be fluid; an office building or partial units of multiplex housing are rented. UMiF receive support for accommodation, medical aid needed for pregnancy and childbirth, and money for childrearing. They also receive services such as vocational training, counseling, and education on childrearing, housework, and cultural education, as well as a partial government subsidy that could be used for childcare services needed when UMiF took vocational training or education.

The treatment of unmarried mothers in Korea is based on traditions of patriarchy [13] and a model to strengthen the role of workers based on capitalist ideology [14]. Specifically, UMiF are asked to achieve two goals during their stay in the facility: raise their children and seek economic independence. However, demands to promote human rights and welfare have increased recently, emphasizing the need for an integrated model of labor and maternal roles to support UMiF [14]. These policies or implementations for UMiF, which emphasize achieving economic independence, are not only inappropriate for raising children, but could also impact their physical and psychological health [13, 15]. The difficulties experienced by UMiF when raising children vary from those of married mothers [14, 16–18], therefore, policies reflecting their unique needs are necessary. This suggests the need for evidence-based intervention based on the UMiF’s unique needs and situation, which would require an in-depth understanding of their real-world experiences. However, studies of unmarried mothers conducted to date have failed to elucidate the advantages, difficulties and detailed experiences of residing in the facilities.

Previous qualitative studies exploring the lived experiences of unmarried mothers have provided an understanding of initial motherhood [19], their parenting-decision processes [20], post-traumatic growth [21], building process and meaning of family [22], and interaction with healthcare providers [23]. As such, these studies focused on identifying specific experiences and processes of decision-making and role-identity changes. Moreover, few studies have been conducted exclusively on facility residents. Therefore, existing literature fails to provide an understanding of the daily childrearing experiences in the specific environmental conditions of a group residence.

It is necessary to investigate the experiences of UMiF concretely to secure practical basic data that can be used for interventions directed at them. Based on this necessity, this qualitative study assessed the experiences of unmarried mothers’ living in facilities with their children.

The methodological principle of the current study was based on the philosophy of hermeneutic phenomenology, which focuses on scientific rigor and validity [24]. Therefore, the results of this study would be reliable for use in supporting UMiF.

## Methods

### Aim

The aim of this phenomenological study is to gain an in-depth understanding of the experiences of unmarried mothers living in facilities with their children.

### Design

This is a phenomenological qualitative study in which qualitative data were collected through individual interviews and analyzed using interpretive phenomenological analysis (IPA) [24] to assess and describe the subjective meaning of the experiences of unmarried mothers rearing their children in facilities in depth.

### Participants

A purposive sampling method was used to recruit participants who shared rich experiences of the phenomenon of interest. The selection criteria were unmarried mothers > 18 years old living in facilities, who raised their children themselves and voluntarily participated in the study. The focus of our study was to investigate the experiences of UMiF while raising their children. The percentage of unmarried mothers considering adoption was high, and the percentage of mothers considering parenting was the lowest in teenage mothers [5]. Unmarried mothers aged eighteen or older, who did not require consent from a legal guardian, were recruited for the study, due to the unique characteristic of unmarried mothers being disconnected from their families [4]. The exclusion criterion was mental impairment, which disabled communication. After obtaining informed written consent and information about participants’ characteristics, interviews were conducted. No participant withdrew from the study. A total of nine unmarried mothers raising their children in four facilities in metropolitan areas were included.

### Data collection

The research team consisted of two nursing professors and four graduate students specialized in mental health nursing and pediatric nursing, who had undergone training in qualitative research methodology and undertaken qualitative research. Among them, two professors and two graduate students conducted face-to-face interviews with UMiF to collect data from May 15 to June 25, 2020. As UMiF raising children were the target sample for this study, participants were recruited in the metropolitan area where 48.7% of the total community residential facilities (19 out of 39) were located and 50% of the residents lived (160 out of 320) [12]. The research team sent out emails to metropolitan community residential facilities to request participation and posted a recruitment announcement in four of the facilities that allowed the recruitment of participants. Interviews were conducted at a preferred time in places such as a counseling room in a residential facility, a cafe near the facilities, or the researcher’s office, depending on when and where the participants felt comfortable. All participants were recruited through research, and there was no prior interaction between the participants and research team except for the interview announcement. Each researcher interviewed one to four participants for one to three hours per session, and eight participants were interviewed once. Due to time restriction and request from the participant, a second interview was conducted on one participant to make up for the unfinished interview guide from the first interview. All interviews were conducted with only the participant and the researcher present except for the fourth participant, who had to take care of her child during the interview. The interviewers revised and modified the interview guide by conducting a role play among themselves in order to improve the quality and consistency of the interview. For all interviews, the transcripts and recordings were shared immediately after their conclusion, and the contents were discussed in regular meetings. The interview guide was revised and modified when needed.

The interview was initiated with an open-ended question, “Could you tell us about your typical day in the facility?”, and the interview continued naturally, according to participants’ answers. The semi-structured interview guide used in this interview consisted of the following questions: “What are the difficulties you face while raising your child?”, and, “What kind of help do you need right now?” Additional questions were asked as necessary to clarify participants’ responses or to probe their words and nonverbal behaviors in detail. All audio-recorded interviews were transcribed by the interviewers themselves. The research team held regular meetings every two weeks to present their analyses and develop themes by comparing similarities and differences between the cases. The recruitment of participants was ceased after verifying data saturation, shown by repeated similarities among data and the absence of new themes. Three participants were shown the transcripts of their interviews and confirmed that the transcripts were consistent with what they had said.

### Rigor

Criteria proposed were used to ensure the validity and quality of the IPA research. First, researchers familiarized themselves with Korean systems and policies related to unmarried mothers to ensure sensitivity to context, and critically reviewed previous studies related to unmarried mothers as well. The research team increased their understanding of study participants through books, movies, and plays related to unmarried mothers, and shared and reflected their own prejudices against unmarried mothers. Additionally, they acquired a deeper understanding of the daily lives and difficulties that unmarried mothers experienced by conducting several studies on unmarried mothers. Such sensitivity to context allowed the researchers to fully empathize with the participants during the process of collecting data, which helped in obtaining rich and reliable interview data. Moreover, purposive sampling was used to select participants with experience, and research results were discussed based on related literature. The validity of the results was secured by confirming the final research results with three participants. Second, we attempted to secure commitment and rigor. Commitment refers to the level of attention and interest in the process of data collection and analysis. A comfortable environment was promoted through sympathetic listening to increase participants’ commitment. Rigor refers to how thoroughly the study was conducted. Data were collected until the data was saturated, and phenomenological reduction and bracketing were performed to prevent distortion of the experience of the participants. Furthermore, meetings between researchers were held regularly to analyze the responses of the participants in a sufficiently interpretative manner, and descriptions and interpretations by each researcher were critically analyzed. Third, the requirements by COREQ (COnsolidated criteria for REporting Qualitative research) [25] were referenced to ensure transparency and coherency in research throughout the entire research process. Furthermore, the COREQ checklist was referenced while writing the manuscript, and the items such as researchers’ characteristics, relationship establishment with participants, and clarity of minor theme were reported without omission. Participation selection, data collection process, interview process, and research analysis process were recorded in detail to ensure transparency. Additionally, coherency was ensured by repeatedly reviewing and modifying parts and the entire manuscript for logical connections between the necessity, objectives, and results of this study. Through these steps, the experiences of UMiF were investigated in a realistic manner, and the gap between theory and existing practices was assessed regarding the phenomenon. Directions for future research and policies were suggested to secure the impact and importance of the study.

### Data analysis

The collected data were analyzed according to the guidelines of IPA by Smith, Flowers, and Larkin [24].

- Reading and re-reading: First, the researchers selected one individual case with experience in the phenomenon and listened to the recorded copy of the interview. The manuscript was read repeatedly to gain a comprehensive understanding of the experience of the participants.
- Initial noting: Exploratory commenting, which recorded the comments of the researchers, was performed. Exploratory commenting includes interpretive and conceptual analysis of the conversations, topic description, and language used by the participants, and the researchers recorded various rich interpretations and questions about participants’ experiences. All researchers performed their own initial noting. The pattern between exploratory commenting sessions was assessed for additional interpretations.
- Developing emergent themes: Initial themes were then derived and recorded based on initial noting. During the regular meeting, the researchers discussed and reached a consensus on the themes they had drawn up.
- Searching for connections across emergent themes: Researchers assessed the relationship between the generated themes and clustered themes.
- Moving to the next cases: After one case was analyzed, researchers moved to another case and repeated the process.
- Looking for patterns across cases: After analyzing other cases, the pattern of recurring themes was identified through analysis between different cases, and the themes were combined.

As a final step, three main themes and eight sub themes were selected, considering the common attributes and individual characteristics of each case. Using this process, we attempted to understand the unique and universal experiences of UMiF.

### Research ethics

During the study, participants’ ethical protection was actively considered. This study was reviewed and approved by the Institutional Review Board (IRB) of the researcher’s institution (IRB No. 2002/001-007). Before the interviews, the research team and the interviewer introduced themselves, the objective and methods of the study were explained to the participants. Ethical considerations such as anonymization and confidentiality of collected data, consent to record and make memos of the interviews, prohibited use of data for other purposes, voluntary participation, and possibility to withdraw from the study, data destruction and absence of any disadvantages resulting from withdrawing, safe storage of research data, and handling of collected data and consent forms were also explained.

## Results

Participants’ mean age was 28.7 years (range 20–37), and the mean age of their children was 19.3 months (range 3–36). One participant was employed, one was a college student, and the others were unemployed, with two participants raising twins (Table 1).

**Table 1:** General characteristics of participants

No	Age	Education	Job	Facility residency length	Age of children	Sex of child	Note
1	24	College Dropout	None	2 years 10 months	1 year 5 month	Female	Twins
2	32	High school	Office worker	3 years	3 year	Female	
3	28	High school	College student	1 year 8 months	1 year 3 month	Female	
4	35	High school	None	2 years 4 months	1 year 6 month	Male	
5	23	High school	None	3 months	3 month	Female	
6	34	High school	None	1 year 1 month	1 year 3 month	Male	Twins
7	20	High school	None	7 months	7 month	Male	
8	25	High school	None	1 year 11 months	2 year 10 month	Male	
9	37	College	None	2 years	2 year 5 month	Female	

The overarching theme found in this study was "a navigation of an ambiguous future with the determination to be a good mother, in spite of facing various difficulties in the limitations of facility life." Three main themes and eight sub themes emerged: (1) adaptation to the identity of “unmarried mother”, (2) willingly undertaking the heavy burden of childrearing, (3) Indispensable but insufficient supports from facilities.

1. Adaptation to the identity of “unmarried mother”

The first theme shows the change of identity experienced by a single woman becoming an “unmarried mother.” The changed identity of the participants can be burdensome as it not only implies transition to motherhood but also additional responsibility as the head of household supporting their children. Social prejudice against unmarried mothers was another factor that challenged their adaption.

1. Bearing responsibility as a head of household

Participants who became the head of household displayed a strong sense of responsibility for protecting the family and childrearing. They endured things they would not have without children. Participants thought that independence was a necessary competency as head of the family, although finishing school and training for work while raising a child was challenging.

*I wouldn’t have been like this if I hadn’t become a mother. I put in extra effort at work because I have a child to support. (Participant 2)*

As shown above, the participants actively dealt with changes due to the responsibility of childrearing that they chose, and accepted such changes as a turning point to start a new life.

*Because of my child, I attended the academy to study and acquired general equivalency diploma… I am living a normal life thanks to my child. (Participant 8)*

2) No longer myself, just an unmarried mother

Participants were treated as solely “a mother of a child” in the facility and were aware that they were expected to live a defined life as a mother obligated to raise her child properly. Although participants voluntarily chose their life as a mother, the obligation that they had to exist only as a mother led to bitterness as they had to put themselves last.

*Everything centers around my child in the facility…(omit)… There is no room for myself. (Participant 8)*

Such bitterness, in contrast to the happiness they experienced outside of the facility where they were treated as “themselves” and not as a “mother,” was strongly observed.

*When I went to vocational training, it was physically exhausting. But I was really happy. What I mean by happy…(omit)… People see me as myself not as a mother of a child. (Participant 8).*

Additionally, participants experienced blatant prejudice and mistreatment against “unmarried mothers” and painfully realized their status as a target of social discrimination.

*They throw insults really loudly so that I can hear, even when the windows are closed. It’s like they torment me on purpose. …(omit)… I guess they abhor this facility… because we are disgusting to them. (Participant 2).*

They felt a lack of justice whenever they experienced social condemnation and were enraged by perpetually negative social perception. However, repeated accumulation of such experiences made them internalize the stigma as unmarried mothers and reevaluate themselves in a negative manner.

*Having a baby is very different from not having a baby…(omit)…When I have a baby, I am all alone in the world… unmarried mother … which becomes a huge handicap and weakness… It's tough. (Participant 2).*

2. Willingly undertaking the heavy burden of childrearing

This theme reveals participants’ experiences of childrearing as UMiF. Raising children made participants think about a mother’s role and led to their self-evaluation as a mother. Although the participants lived in a community residential facility, the reality that they were the only ones taking care of their children was harsh and painful. They also felt sorry for their children due to the absence of a father, which their extra efforts could not compensate for. However, as they raised their children, they gained comfort from them, and they held hope for the future while going through the challenges of job training and preparing for independence.

1. An unstable journey towards being a “good mother”

Participants generally had an “image of an ideal mother,” which served as a guide for their mothering. However, they perceived an appreciable gap between the ideal mother and themselves. Moreover, participants also lost confidence and faced pressure from the negative self-evaluation of their qualities as mothers.

*I am sorry to my child. I do not think I am being good to her, I am sorry that I feel like a bad mother… We say that a lot. (Participant 7)*

*Am I depriving my child of an opportunity to be raised by better parents who are happier than me?…(omit)… What if I am taking away my child’s right to be happy just because I’m the mother. (Participant 6).*

Participants poorly evaluated themselves as mothers. They seemed to consider their own family as the root for their lack of ability. Participants felt they needed to be psychologically stable to avoid passing on their negative childhood experiences to their children. In particular, mothers believed that their actions and qualities had great effects on their child's emotions and personality development. Thus, they considered the quality of their relationship with their children as an important factor in the challenging situations they experienced.

*I told you about my upbringing. I grew up with such a bad family background…(omit)…sometimes I’m nervously careless to my child. (Participant 3)*

*I am more unstable in my mind than my ability worrying about because of this instability… it directly affects my kids. It has enormous effects on the mind of my babies. As my kids grow up, all because of me… ruin my babies. What if my babies' lives are ruined? That is what I fear a lot. (Participant 1).*

*Minimum responsibility as a parent. I failed to receive that when I was young, and I don’t want that for my child. I want my child to grow up to be happy. (Participant 4)*

2) A lonely journey of childrearing despite living with others

Participants experienced the hardship of raising children on their own in everyday life. UMiF, who were already overwhelmed by parenting, struggled to be independent. Being independent is essential for UMiF; however, the time they could spend with their children gradually decreased as they prepared to be self-reliant, making mothers feel unfortunate and uneasy. Particularly, participants faced the greatest difficulty when they or their children fell sick and had to be hospitalized. As most participants had no alternatives other than stopping their economic activities or postponing their children’s surgical treatment, they were thoroughly aware that they had to endure crises in life alone.

*When I finish my daily tasks at 5 and head home, I have very little time. I run to pick up my child, and once we get home, I cannot rest. When we get home, my child is hungry and cries. So, I give a little bit of food to calm my baby down, and I quickly cook dinner. (Participant 2).*

*The baby survived on antibiotics. I couldn't hospitalize her due to work, so I had no choice but to keep giving antibiotics. Sometimes for a month, or even two. (Participant 2)*

Other residents in the same facility could not be relied on when help was needed. This was because they were not only busy taking care of their own children, but also indifferent towards others’ lives or their parenting, and were nothing but “coresidents.”

*When the kids are together, all the mothers are busy taking care of their own children. The next-door mom is very nice to her own child, but whenever my child approaches them she avoids him and calls me to take him. (Participant 4).*

3) Absence of father, which cannot be filled

The majority of participants perceived that the absence of a father was a deficit that they had to fill. Such thoughts motivated the participants to work harder, and they undertook the burden of the role of the father as well. However, through the behavior of their kids, participants realized that the absence of a father could not be resolved by simply playing his role. This became a factor that led to mothers feeling sorry for their children.

*There was a story involving “dad” and my child just ran away while eating. She was hiding in the tent crying…(omit)… It really hurts. (Participant 2)*

4) Growth of a child which provides comfort and cheer.

Participants living a tough life were comforted by their children. The participants stated that they felt proud as they witnessed their children crawl, speak, laugh, and express affection. Their children were the driving force for participants to tolerate their harsh reality and hope for a better future.

*When my child enjoys the meal I prepare, it makes me forget how exhausted I am and I feel proud. (Participant 7)*

*As I get to communicate with my child I feel more responsible…(omit)… It makes me happy to think about our future. Although things are hard right now, who knows if I can be successful in the future… no one knows…(omit)… I feel happy imagining our lives together after my child grows up. (Participant 8).*

3. Indispensable but insufficient supports from facilities

This theme covers the participants’ experience of receiving aid provided by the facilities. The facilities, as the only places that offered help to UMiF who had nothing to rely on, were like lifesavers to them and their children. However, the support provided by facilities only enabled survival and was did not meet the needs of UMiF and their children. The dissatisfaction with support from their facilities caused anxiety and psychological pressure in participants.

1. A place of mercy that aids survival and parenting

The participants decided to have children and raise them themselves; however, they were disconnected from the child’s father and their family. Thus, they lacked a place to raise their children and the capacity to make a living. These UMiF were grateful for the facility that provided them with residence and material support. The participants, who became mothers without any preparation, recognized that the parenting knowledge they were taught, and their time in the facilities, helped them fulfil their roles as mothers.

*In the community, I have never received any help. At first, I did not know what to do because I was grateful to the facility. (Participant 1)*

*When things were really tough because I had nowhere to go…as if I’m holding onto a lifeline… the facility gave me a chance for life. I’m really grateful for that. (Participant 6)*

*I learn about baby food and get a counselling on raising children here. I keep learning. (Participant 7)*

2) Facilities that don’t always meet mothers’ and children’s needs

Support provided by the facility did not satisfy the expectations of UMiF, as the management policy of the facilities did not fully reflect the participants’ desire to raise their children well. Extending their stay at the facilities required vocational training or studies, which led to participants sacrificing time with their children and focusing on satisfying the requirements. The participants believed that it was unreasonable to lose their parenting time to meet the conditions of residence in the facility as they had entered the facility to raise their children properly. However, the participants, who had no other options, understood that satisfying the requirements was not a choice, but a necessity.

*Going to school or receiving vocational training for economic independence is a requirement to stay in this facility…(omit)… because help is provided under such condition, everyone sends their kids to daycare before they become one-year-old to receive vocational training, even though they want to raise their children by themselves. (Participant 4).*

The participants needed a place to stay until they were fully independent and ready to support their children on their own. However, the requirements to stay at the facilities, and the regulation in terms of residence did not fully reflect the residents’ preparedness for independence or their desire of parenting. Therefore, participants were constantly concerned and faced pressure to locate a place to live with their children after their time in the facility. Participants extended their stay as much as possible by satisfying their residence conditions. However, the facility as a home has an expiry date. Thus, most UMiF who did not have realistic solutions felt pressured. Participants attempted to be independent through the Successful Employment Service Package (SESP); however, employment was not guaranteed. Moreover, the UMiF were financially unstable, regardless of if they were receiving basic living expenses or had occupations. Therefore, living with their children outside the facility, where support would be lost, presented an uncertain and worrisome future, including inevitable poverty, for UMiF.

*After paying for rent, taxes, health insurance for my child, and living expenses, my salary runs out and I don’t even have 100,000 won to spare… It made me realize I wouldn’t have any large sum of money when my child gets sick. Things will be rough without the help from facility… (Participant 2).*

The physical conditions of the facilities were unsatisfactory, and only provided the bare minimum for survival. A majority of participants shared rooms with other residents, as well as essential spaces such as the living room, kitchen, and bathroom. Sharing rooms in regular homes that were not designed for communal living made leading an independent life impossible for UMiF. Daily life in facilities entails limited space and sharing resources with other residents. Order and distribution were prioritized in such a manner that activities like giving showers to and feeding their children could not be performed freely.

*Other residents have to cook and feed their children… I also cannot just stand on the side and keep rushing them in the kitchen… I was able to wash the baby bottle at 8 and 9 o'clock at night. Waiting for next time… Anyways, such things were annoying. (Participant 4).*

Some participants believed that their children were negatively influenced by other UMiF and their children living in the common space. However, they understood this to be a natural problem, stemming from communal living itself. Thus, UMiF coped with such problems patiently rather than attempting to improve them. Participants experienced dissatisfaction with communal living facilities and anxiety while acknowledging that the parenting environment could not be controlled educationally. Moreover, communal living, where privacy is not guaranteed and personal space is lacking, led to greater perception of “myself” not receiving respect.

*My child was not used to be like that. However, starting at one point, my child closes the door and stays in the room once he comes home. My child starts yelling whenever the next-door kid who annoys him tries to touch his toy. (Participant 8).*

*Another mother in the facility does not say hi to others and displays poor manners. I don’t think mothers should have such poor disposition that should not be modelled by their children. (Participant 6).*

Their dissatisfaction with services provided by the facilities also reflected in the programs and services offered. Participants experienced their desperate needs being undermined by the goal of childrearing when the facility worker didn’t respond seriously to their request for help with psychological difficulties. They considered the education and services provided by the facility as short-term and customary and, therefore, ineffective for their actual needs.

*Instead of giving me a referral or paying closer attention to treat my postpartum depression, the staff just ask me “what are you going to do with the depression medicine?”…(omit)… They seem to think our depression is not a real one. They don’t take it seriously. (Participant 6).*

*I don’t know, it’s just temporary. All the counseling is only for the moment and nothing really changes afterwards…(omit)… maybe because it wasn’t for long term. I feel good when I receive counseling, but everything’s the same after it’s over. (Participant 9).*

## Discussion

This study investigated the lived experiences of UMiF. Three main themes were derived from the results: (1) adaptation to the identity of “unmarried mother,” (2) willingly undertaking the heavy burden of childrearing, (3) indispensable but insufficient supports from facilities.

The first theme, “adaptation to the identity of ‘unmarried mother’”, showed the acceptance as well as resistance displayed by participants regarding their changed identity. The process of pregnancy, childbirth, and childrearing is a transition accompanying the establishment of a new identity as a mother, and it is important to receive support from one’s family, partner, friends, and local community to achieve successful transition [26, 27]. However, UMiF, who are disconnected from their family, child’s father, and relatives and live in a facility, cannot rely on such support, and therefore their adaptation is even more challenging. Additionally, for UMiF, this process not only implied a transition to motherhood but also to a head of household in charge of supporting the family. Hence, far more energy was needed for adaption. Furthermore, the social prejudice they face during this process increases their difficulties. Such a multitude of hardships interfere with their transition to a head of household and mother, lowering their confidence and self-esteem. This can ultimately become a threat to the survival and health of their families. In addition to offering essential help needed for survival, the support provided by facilities should be extended to connecting the UMiF to local societies and communities where they can form positive interpersonal relationships and gain proper social support.

UMiF perceived that “they” no longer existed in the front lines of their lives but were rather pushed to the background by another identity. Such feelings result from experiencing the altered social position of “unmarried mothers” who experience prejudice and discrimination outside the residential facilities. Community prejudice and discrimination are factors that affect depression in unmarried mothers [10], and experiencing stigma more intensely increases their tendency to internalize it [28]. In fact, the “I,” who is “left out,” may experience anger and depression due to disrespect. However, internalized stigma degrades self-esteem and self-efficacy [29], preventing them from expressing this negative experience or demanding fair treatment. Lower self-esteem can increase the likelihood of experiencing depression and parenting stress and can reduce the self-efficacy of parenting [18]; therefore, the therapeutic intervention of internalized stigma is required to help UMiF’s mental health and childrearing.

The theme “willingly undertaking the heavy burden of childrearing” shows the experiences of UMiF who are discouraged by the harsh reality they encounter while raising their children, but find joy in watching their children grow and try their best to pursue a better future for them. UMiF were discouraged by self-disappointment and prejudice toward unmarried mothers despite their efforts at good parenting. This finding is consistent with previous studies, indicating that an “ideal motherly image,” which may be a suitable method for unmarried mothers to acquire knowledge, skills, and attitudes necessary for childrearing [30] may lead to conflicts in roles when expectations are not met [31]. UMiF were highly motivated to be good mothers. Therefore, parenting education and counseling interventions to relieve their negative self-evaluation and burden of decreased maternal confidence need to be provided.

In our study, UMiF thought that their psychological health had greater effects on child rearing than independence and employment. Moreover, like Kwon [32], participants were concerned that their negative experiences of family relationships in the past would be internalized and stigmatized into an undesirable parenting attitude and adversely affect their children. This finding indicates that UMiF are determined to raise their children properly, unlike their own upbringing. Therefore, it is necessary to provide a long-term intervention for psychological healing and mental health that can meet the demands of UMiF.

The theme “a lonely journey of childrearing despite living with others” shows a glimpse of the unique climate in facilities where residents who share communal living space do not help each other and live uncooperatively. This is likely due to emotional instability experienced by UMiF who live life anxiously every day, and lack peace of mind. Moreover, as shown by the statements of participants, they could not build faith in their child’s father and were not accepted by their own family. Therefore, it was likely that they lacked interpersonal relationship skills. Hence, in addition to instrumental support, functional support through the philosophy of community management is also needed in facilities for UMiF. Offering education that can foster a sense of community [33] will help the residents cooperate with each other and contribute to communal living.

The UMiF considered their children as an opportunity and motivation for positive changes in themselves. This observation was also reported in a previous study [34] that demonstrated that the children’s growth was rewarding and acted as a driving force for unmarried mothers. Positive emotions felt towards their children is a factor that positively affects their transition to motherhood [35]. Such a phenomenon can be interpreted as an alteration of their previously negative identity into a positive one through a transition to motherhood. Unmarried mothers believed that their “refracted life” [36] and improper behaviors [37] caused them to become unmarried mothers, and participants in this study were also seen as satisfied with the changes they observed in themselves while becoming a good mother.

Our study showed that support from facilities was an absolute necessity, but could not meet the needs of UMiF and their children. UMiF felt sorry for their children and were concerned about the negative consequences due to a lack of parenting time caused by vocational training. Anxiety, even among married working mothers, is also caused by a lack of parenting time for children [38]. This is in line with a previous study, which reported that facilities mostly control the lives of UMiF instead of providing sufficient childcare [39]. Due to the lack of social support and resources, material assistance and emotional support, as well as childcare services, are extremely important [40] for UMiF while they stay in facilities. Therefore, emotional support and material assistance should be sufficiently provided in the facilities, as well as the childcare services that allow UMiF to leave for vocational training without concern. But unlike unmarried mothers, married working mothers believed that their children would understand and be proud of their occupation [41]. This can be attributed to the fact that the lack of parenting time comes from the inevitable choice of the participants to meet the requirements for staying in the facility, and that their attempt to find a job did not guarantee absolute independence. While raising children, unmarried mothers search for jobs that allow room for parenting. The participants of our study were also going through vocational training, making the best efforts to achieve both independence and parenting goals. The uncertainty of a newly acquired job and low vocational self-efficacy can interfere with their achievement of independence, which serves as their most significant goal. This may explain the differing perceptions between unmarried mothers and married working mothers regarding work. Nonetheless, participants hoped to raise their children independently, while enduring hardships, and actively seeking jobs. The results of this study confirmed the finding that unmarried mothers prepare for a promising future even in the frustrating situations they experience in the facilities [37, 42]. However, UMiF must acquire the necessary basic requirements for job opportunities, which distinguishes their situation from that of previously working mothers who are preparing to return to their prenatal occupations. Therefore, preparing for independence in UMiF may require long-term socioeconomic support.

The participants imagined their life after they left the facility, where they were only permitted to remain for a limited time. Although UMiF could predict realistic problems that must be overcome, they faced uncertainty, as they lacked solutions to those problems. Many UMiF, who have no family to rely on and no reliable source of support, feel severe instability and fear. Many participants were unemployed or had quit their job before entering the facilities, and decided to live in these residential facilities as they lacked economic independence. Thus, employment is an uncertain challenge that requires tremendous effort. In fact, most UMiF fail to find a job, which leads to poverty after leaving the facility [10]. Therefore, we suggest systematic and long-term support to ensure independence of UMiF, in addition to economic support for a certain period after the expiration of their stay in the facilities. Furthermore, their departure from the facilities should be determined not by the duration of residence, but by the evaluation of their preparedness for independence.

Discomfort in communal life and conflicts with co-residents were observed among UMiF, contrary to the findings that communal life is beneficial for the growth and learning of considerate thinking in children [43]. In particular, our study demonstrated that securing personal space in residential facilities is a factor that greatly affects resident satisfaction [44]. Therefore, it is necessary to organize and operate residential facilities that reflect the needs of UMiF.

Practical support tailored to the desperate needs of UMiF, rather than customary and unilateral programs that do not represent their needs, are necessary. Interventions that can help UMiF gain the strength to be independent are essential for those worried that their psychological instability may negatively affect their children. Furthermore, stress from communal living likely increased during the coronavirus disease-19 (COVID-19) pandemic, where communication with the outside world was highly limited. It is thought that anxiety caused by COVID-19 [45], in addition to maternal anxiety [46], would have negative effects on childrearing stress among UMiF. Hence, creative and thoughtful measures to overcome such problems are required.

Negative social perception of unmarried mothers is not restricted to Korea, and these residential facilities for unmarried mothers are operated by various entities in a diverse manner in numerous countries across the world, including the United States, England, and Malaysia [47–49]. Although our study is limited to the childrearing experience of unmarried mothers residing in government-sponsored facilities in Korea, it elucidates pros and cons of the support provided by these facilities in addition to the lived experiences of UMiF who have nowhere else to go. Our study investigated the experiences of unmarried mothers, who belong to a vulnerable social group and are the target of social stigma, raising children in temporary residential facilities, in depth; hence, we believe that our study highlights universal experiences of UMiF that transcend cultural and circumstantial boundaries.

In this study, we observed that UMiF struggled with childrearing and independence. However, they were willing to become good mothers and sought a brighter future for their children while attempting to be self-reliant. The results of this study correspond to the practical challenges experienced by UMiF, which are meaningful because they reflect the service needs of UMiF. The results can be applied in practice as follows: First, it can be used as basic information for creating tailored interventions centered on the needs of UMiF. Second, it could serve as the foundation for the development of assessment scales that can evaluate their needs or service satisfaction. Third, it can be used as educational material for facility workers to provide an understanding of the experiences of UMiF from their perspective.

This study has some limitations. The study was conducted after the spread of COVID-19 in an environment where several programs and services provided in facilities for unmarried mothers were limited. Several participants lived in an environment where visits from outsiders, group gatherings, and certain outdoor activities were restricted, which could have positively or negatively affected their perception and experience of childrearing.

## Conclusion

This study explored the experiences of UMiF with their children and the meaning of such experiences in depth. UMiF tried their best to become good mothers, as well as heads of household that could provide for their family while preparing themselves to be economically independent, and interpreted such changes as a positive turning point of their life. Meanwhile, they also experienced injustice stemming from social prejudice as well as a sense of loss from identity change. While parenting was a joy and offered a driving force for self-growth, it brought more challenges and struggles to UMiF such as low maternal self-esteem, difficulty in preparing themselves for economic independence while raising children, and feeling sorry for their children due to the absence of a father. Support from facilities was essential for those who had nowhere to go and were unable to make a living, but did not suffice to meet their goals of becoming a better mother and pursuing hope for the better future. The findings of this study reflect the necessity of support for UMiF, and shows that they need a wide range of resources for parenting and independence, intervention programs for psychological health, connection with community resources that can socially support them, and living spaces that reflect their needs.

## Data Availability

The datasets generated and analyzed during the current study are not publicly available as the data contains sensitive, detailed, and personal experiences of vulnerable participants. Research data may be available upon request through the corresponding author after obtaining additional approval from the Institutional Review Board.

## Abbreviation

UMiF: Unmarried mothers who live in residential facilities
